# Supplementary material for: Day 100 Recovery of Absolute Number of Inhibitory KIR2DL2 and Activating NKp30 Natural Killer Cells Predicts Survival Post-Autologous Stem Cell Transplantation in Lymphomas
Source: Biomedicines. 2024 Aug 9;12(8):1808. doi: 10.3390/biomedicines12081808 (PMC11351217; doi:10.3390/biomedicines12081808)
Supplement: Supplementary file 1 [file biomedicines-12-01808-s001.zip › biomedicines-3124896-supplementary.pdf]

A

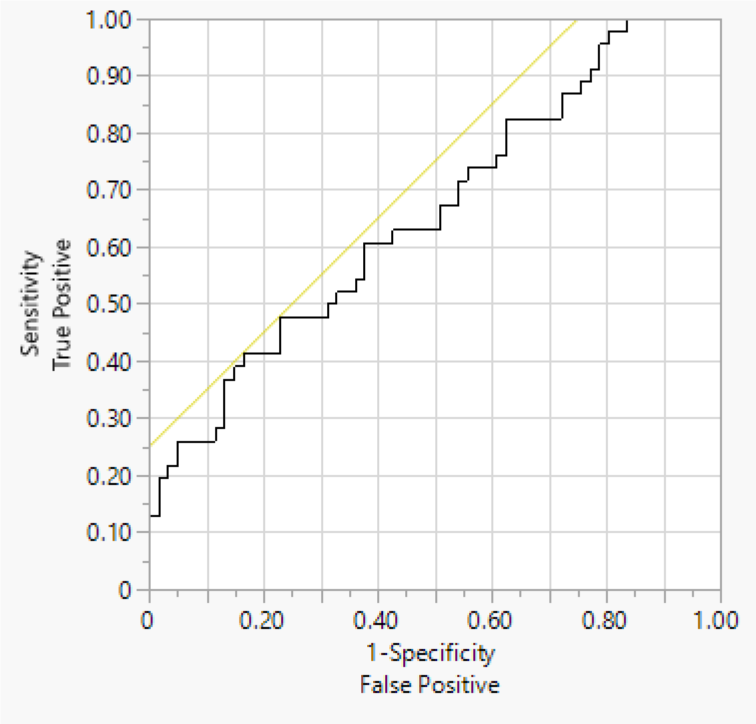

B

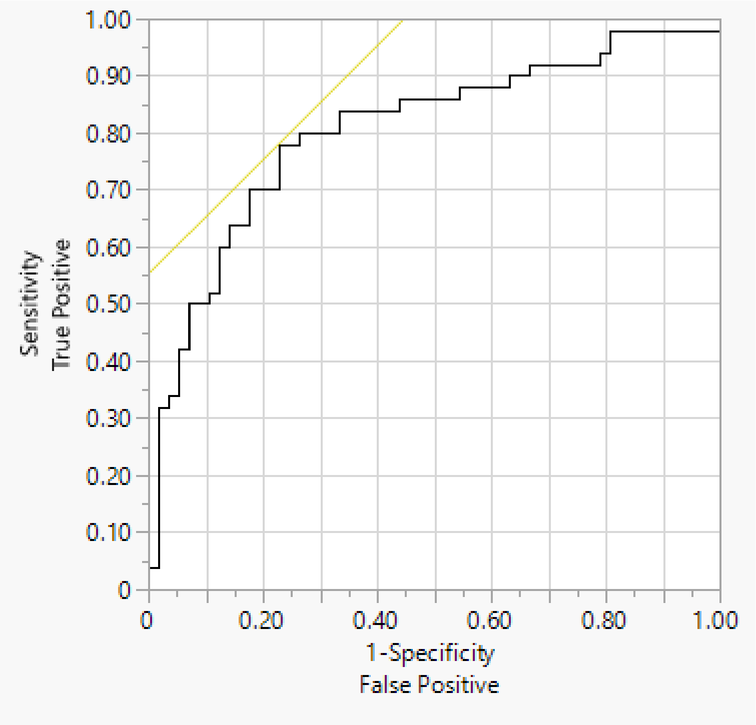

KIR2DL2
AUC = 0.66
P < 0.01
Sensitivity = 61%
Specificity = 56%

NKp30
AUC = 0.81
P < 0.0001
Sensitivity = 78%
Specificity = 77%

Figure 1, supplemental

A

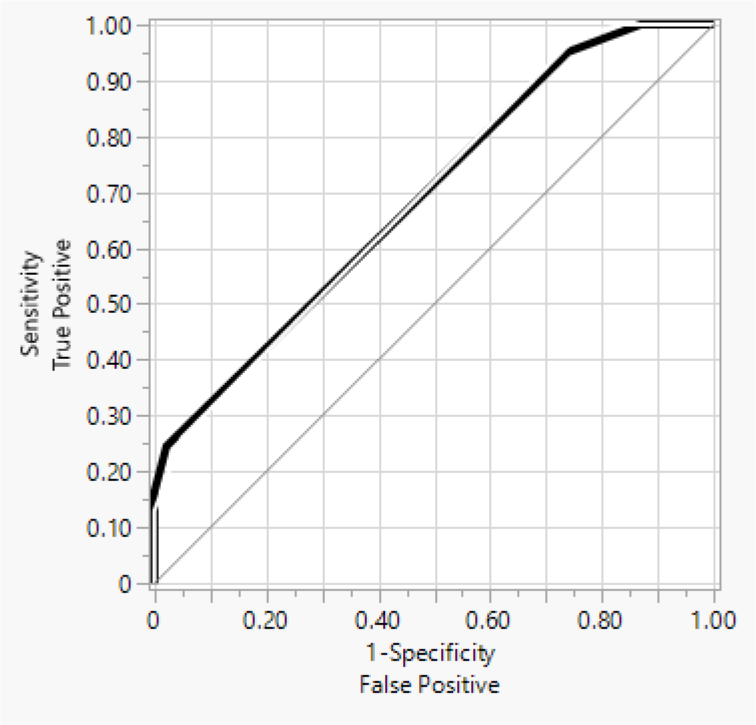

B

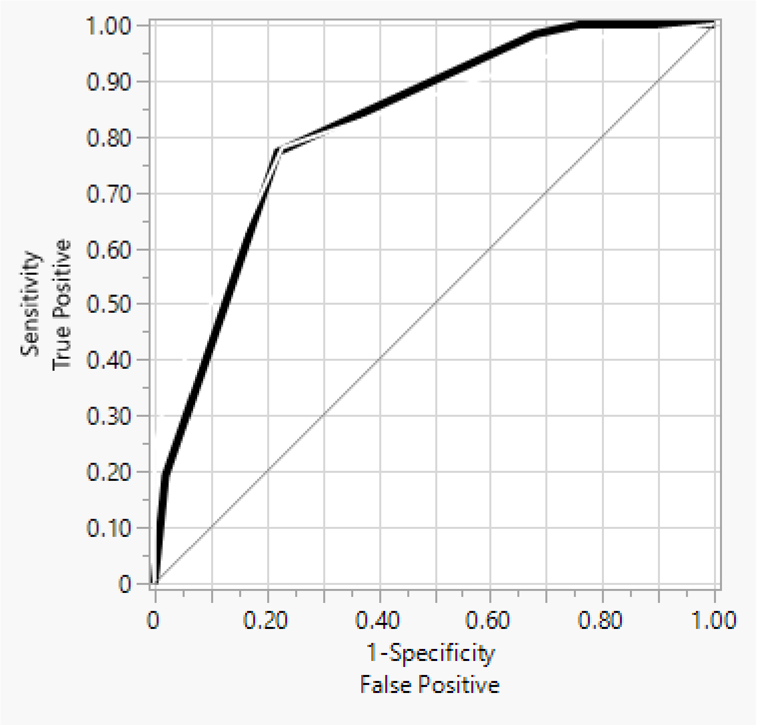

KIR2DL2
AUC = 0.66

NKp30
AUC = 0.82

Figure 2, supplemental

Day 100 PFS KIR2DL2

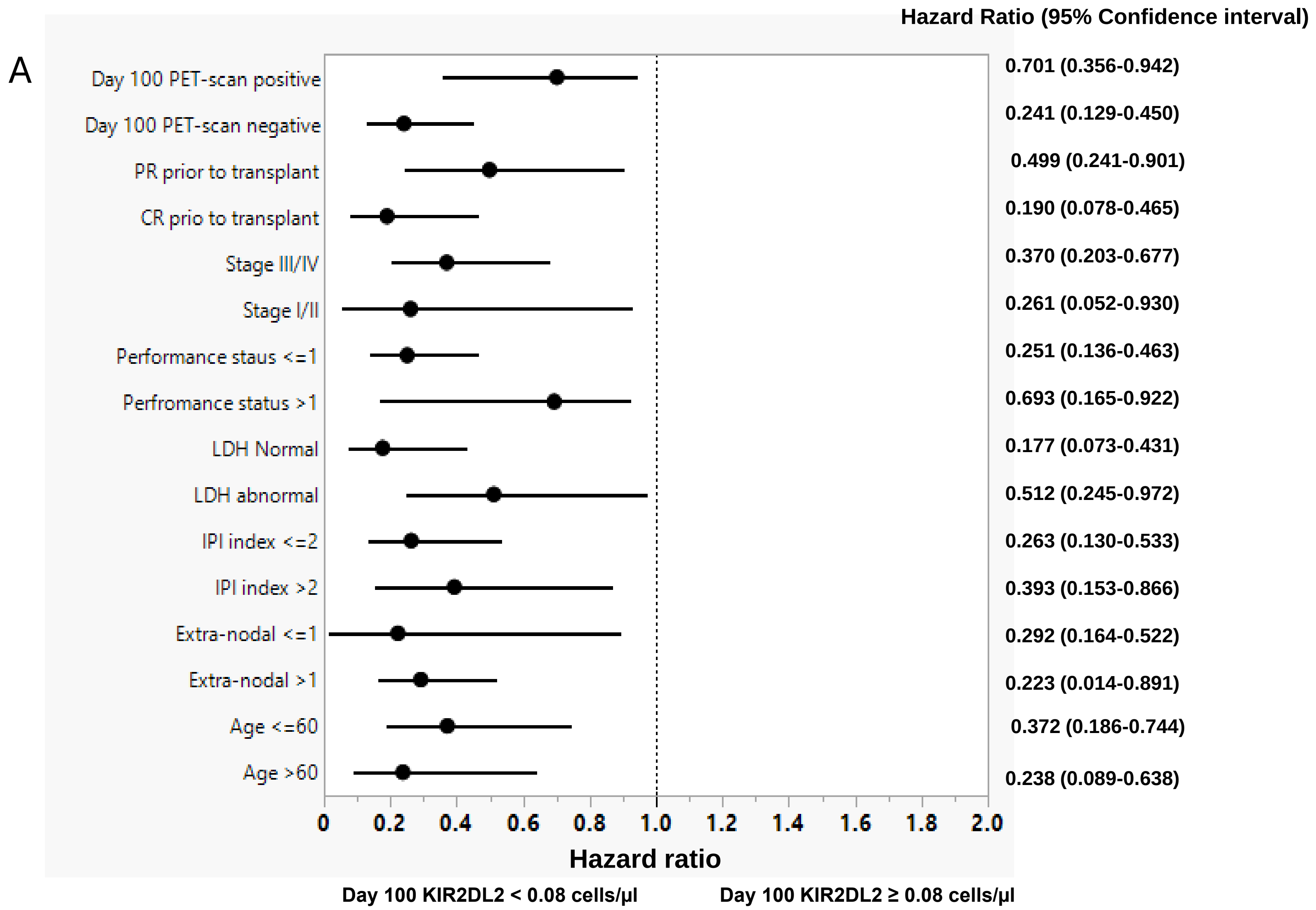

Figure 3, supplemental

Day 100 OS NKp30

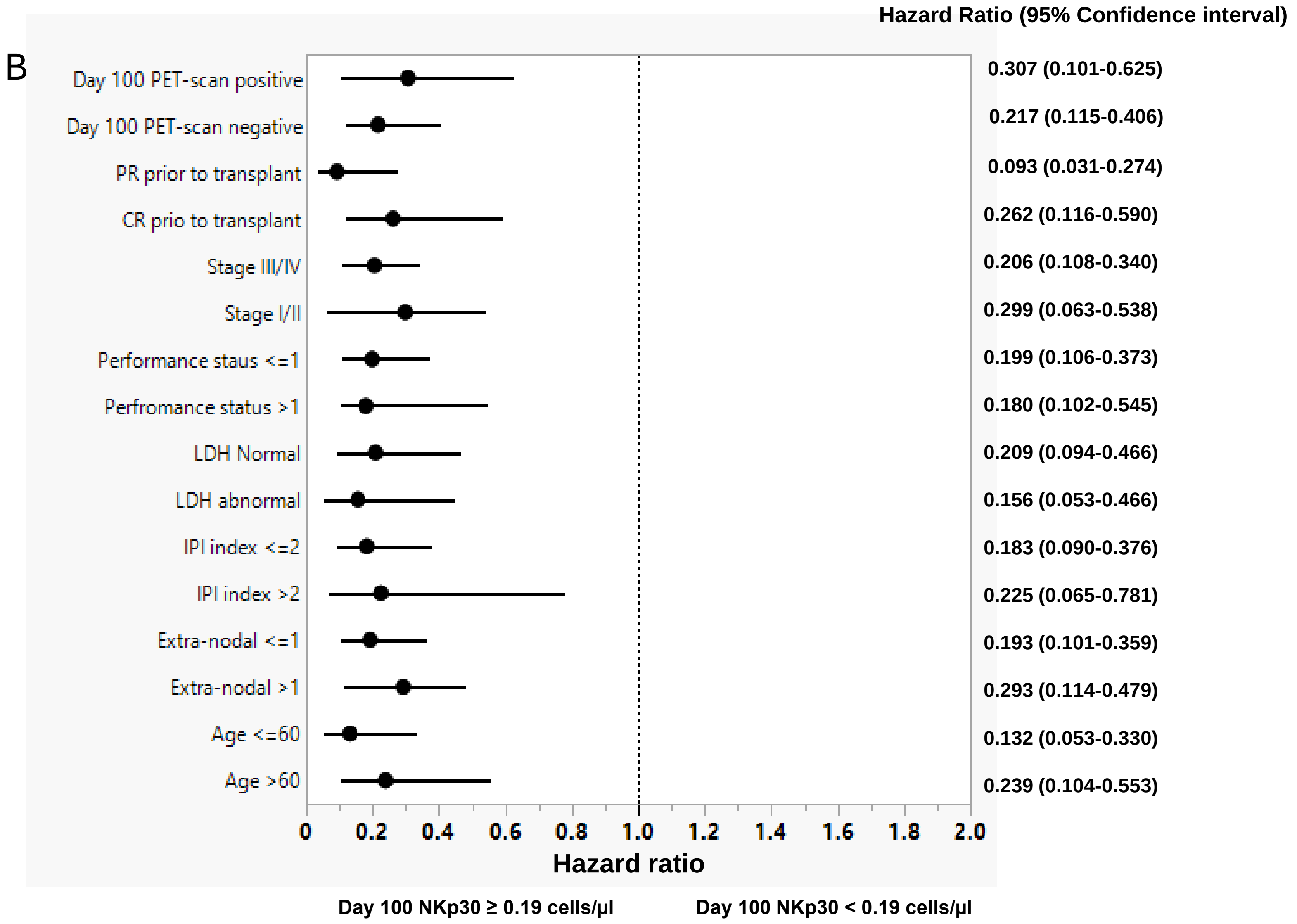

Figure 3, supplemental

Day 100 PFS NKp30

C

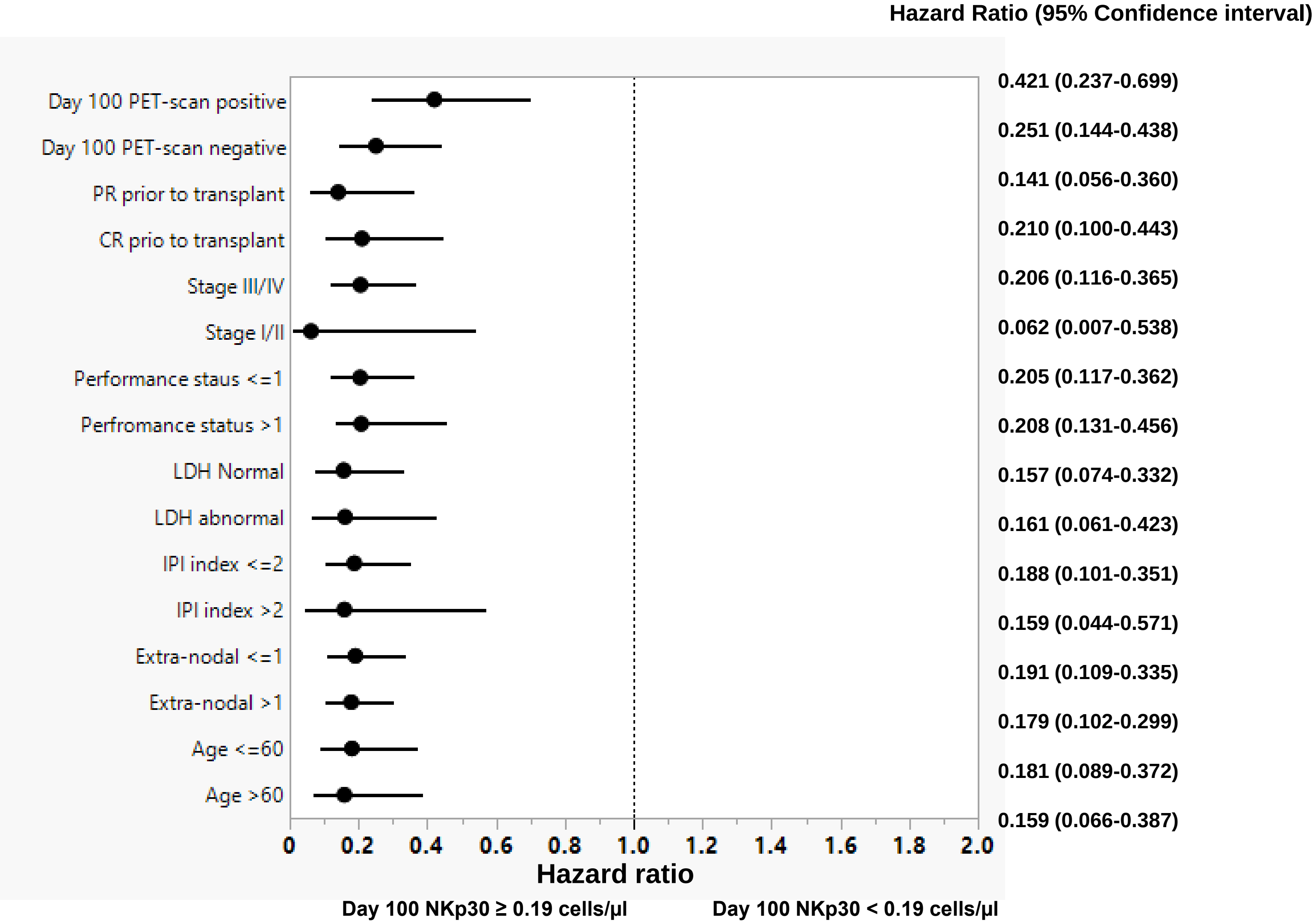

Figure 3, supplemental
